# Clinical Summaries of Social Media Timelines for Mental Health Monitoring: Human Versus Large Language Model Comparative Evaluation Study

**DOI:** 10.2196/71230

**Published:** 2026-03-27

**Authors:** Ayal Klein, Jiayu Song, Jenny Chim, Liran Keren, Andreas Triantafyllopoulos, Björn Schuller, Maria Liakata, Dana Atzil-Slonim

**Affiliations:** 1 School of Electronic Engineering and Computer Science Queen Mary University of London London, England United Kingdom; 2 Department of Psychology Bar-Ilan University Ramat Gan Israel; 3 Department of Computer Science Ariel University Ariel, Judea and Samaria Area Israel; 4 Chair of Health Informatics Technical University of Munich Munich Germany; 5 GLAM – Group on Language, Audio, & Music Imperial College London London, England United Kingdom; 6 The Alan Turing Institute London United Kingdom

**Keywords:** social media, monitoring mental health, clinical summaries, natural language processing, large language models

## Abstract

**Background:**

Social media timelines contain rich signals of users’ mental states but are too voluminous for direct clinical review. Although large language models (LLMs) demonstrate robust linguistic and summarization capabilities in general‑purpose tasks, distilling clinically relevant insights demands deeper psychological analysis and sensitivity to each individual’s unique personality and context. Accurately capturing subtle, personalized affective and behavioral patterns remains a significant challenge for current models. A thorough, systematic evaluation of LLM‑generated clinical summaries is therefore essential to understand their readiness for real‑world mental health monitoring.

**Objective:**

This study evaluates the ability of an LLM-based pipeline to generate clinically meaningful summaries of social media timelines, compared to summaries written by human clinicians. The summaries are structured along 3 key clinical aspects, including an overall mental health assessment, intrapersonal and interpersonal patterns, and mental state changes over time.

**Methods:**

We use a recent state-of-the-art approach that combines a hierarchical variational autoencoder (VAE) with an LLM (Large Language Model-Meta AI 2 13-billion-parameter version; LLaMA2 13B). This method first summarizes the patient’s history using the VAE and then transforms this summary into a clinical narrative using the LLM. We also test both single-step and multistep LLM-prompting techniques and devise comprehensive clinical prompts. For 30 social media timelines, model outputs were evaluated against human-written summaries through human ratings and expert qualitative analysis. Linguistic diversity was automatically measured as a proxy for personalization.

**Results:**

Human summaries scored highest for factual consistency (3.75) and general usefulness (3.63). The timeline-hierarchical variational autoencoder (TH-VAE) model outperformed LLaMA for factual consistency (3.35 vs 3.08) and general usefulness (3.28 vs 3.38). Both 2-step models were comparable to humans in describing interpersonal and intrapersonal patterns (3.45-3.48 vs 3.33) and changes over time (3.42 vs 3.35-3.30). The naive LLaMA baseline scored lower on all criteria except factual consistency. Furthermore, a qualitative analysis observed that human summaries provided more accurate, deep, and personalized insights, while LLMs offered more exhaustive but generic descriptions. Quantitatively, linguistic diversity was higher in human summaries both at the semantic level (mean Cohen *d*=1.19) and at the surface level (mean Cohen *d*=1.31).

**Conclusions:**

At this time medium-size LLMs can generate largely accurate and informative clinical summaries of social media timelines, and advanced prompting boosts performance modestly. However, at the time of this writing, they underperform human clinicians in capturing subtle psychological nuances and individual idiosyncrasies. Future work should integrate domain‑specific fine‑tuning and enhanced context modeling to improve LLM clinical fidelity.

## Introduction

Social media platforms have emerged as a window into individuals’ inner lives, providing a rich source of information about their thoughts, emotions, and experiences. These vast, unstructured data hold immense promise for enhancing mental health monitoring and support, but their sheer volume and complexity pose challenges for clinicians seeking to extract meaningful insights. This study investigated how advanced artificial intelligence (AI), specifically large language models (LLMs)—powerful AI algorithms trained on large amounts of textual data—can be leveraged to distill this wealth of data into concise, clinically relevant summaries.

Current methods for assessing mental health primarily depend on self-reports completed by patients or structured interviews conducted by clinicians. While these methods are essential in mental health research and practice, they have notable limitations. Self-report measures can be influenced by limited self-awareness, reluctance to complete questionnaires, and the constraints of predefined response options [1]. Clinician assessments, though valuable for their expertise, tend to be time-intensive, expensive, and reliant on patients proactively seeking help, factors that can hinder early detection and intervention. In addition, these methods often provide a static snapshot of an individual’s mental state and fail to capture its dynamic and fluctuating nature [2]. Leading researchers have highlighted the urgent need for innovative assessment methods that can better track these dynamic shifts to enhance monitoring and prevention efforts [3].

In recent years, social media has emerged as a particularly promising resource. With appropriate safeguards and informed consent, it provides a continuous stream of self-reported data and interactions that could complement traditional mental health assessments [4]. Posts often reflect emotional states, relationships, and life events, potentially yielding valuable insights into an individual’s well-being. However, the sheer volume and unstructured nature of social media data present challenges for clinical application. The noise of irrelevant content and the complexities of online self-expression make it difficult to discern meaningful patterns and track changes in mental state over time.

Clinically focused summaries of social media timelines could address these challenges by synthesizing the most pertinent information into a coherent narrative. Such summaries, capable of capturing fluctuations in mental states and highlighting key clinical concepts, have the potential to enhance clinical understanding, support ongoing monitoring, and aid in the early detection and prevention of mental health issues. By efficiently presenting relevant information, these summaries could augment clinicians’ capacity to provide timely and effective care.

The emergence of powerful language technologies, particularly LLMs, offers new avenues for summarizing clinically relevant information from social media timelines. Trained on massive datasets, LLMs can capture complex linguistic patterns and generate fluent, context-sensitive outputs [5-7]. Their generative abilities build on decades of progress in text summarization, which has advanced from classic extractive approaches [8,9] to neural extractive models [10] and modern pretrained sequence-to-sequence abstractive methods [11]. These developments have paved the way for applications in specialized domains, where summarization is required to handle diverse and noisy inputs.

Recent research has applied LLM-based summarization to a range of clinical contexts, including electronic health records [12], clinical trial reports [13], therapy sessions [14], and mental health forum posts [15]. Beyond summarization, LLMs have also been used to generate structured medical notes from physician-patient encounters [16], condense electronic health records [17], and extract key information from medical texts while addressing hallucination risks [18]. They have further been explored for tasks such as triage, diagnostic support, and even simulated therapy dialogue, highlighting both their promise and the need for rigorous evaluation in mental health settings.

In parallel, computational mental health studies confirm the effectiveness of AI in analyzing social media data with user consent for support purposes [19-22]. However, most of these approaches have centered on classification, detecting signals of depression, anxiety, or suicidal ideation using supervised learning and engineered features such as n-grams, linguistic inquiry and word count categories [23], and posting behavior. While valuable for large-scale risk detection, classification methods often overlook the contextual richness of individuals’ timelines and do not produce coherent, human-readable accounts of lived experience. By contrast, generative tasks such as timeline summarization may capture evolving trajectories of thoughts, emotions, and behaviors, promoting a more comprehensive and bottom-up understanding of mental health [24-27].

Despite their promise, LLMs face key challenges when applied to mental health summarization. Ensuring factual accuracy is crucial in clinical contexts, as models may generate inaccurate or misleading information [28]. Modeling temporal dynamics is also difficult; LLMs are typically optimized for short-range coherence and may miss subtle emotional changes across time [29-31]. Moreover, current models often struggle with personalization, defaulting to generic phrasing and failing to reflect an individual’s unique expressive style, interpersonal context, or situational nuance [32]. Finally, the clinical integration of LLMs must proceed cautiously, with attention to safety, fairness, and reliability, including robust anonymization and safeguards against bias to avoid perpetuating disparities [24-26]. Successful implementation requires close collaboration between clinicians and AI researchers to ensure that models are shaped by clinical reasoning, grounded in psychological theory, and responsive to patient welfare [33].

Thus, creating social media timeline summaries that accurately capture individuals’ mental states and dynamics remains a challenging yet crucial goal. To advance this effort, this study investigates the potential of advanced LLM-based methods in generating clinically insightful summaries from social media timelines and evaluates their quality as compared to clinicians’ summaries. We implement both straightforward prompting techniques as well as a recent approach that incorporates unsupervised abstractive summarization through a hierarchical variational autoencoder (VAE) [34]. To analyze the quality of the generated summaries—ensuring they are factually accurate, include all significant information from the timeline, and capture the unique characteristics of the patient—we conduct a comprehensive evaluation involving quantitative comparison of human ratings, qualitative expert analysis, and computational metrics.

The aim of this study is thus to evaluate whether LLM-generated social media summaries can approximate the clinical quality of human-written ones, thereby assessing their feasibility as tools for augmenting mental health monitoring.

## Methods

### Dataset

We use the TalkLife dataset [35], which comprises 500 user timelines from the TalkLife platform (TalkLife Ltd). The timelines, which vary in length (12-124 posts, average 35) and time span, have been manually pseudonymized to remove personal names and places.

This dataset is preannotated with moments of change [35], denoting sudden mood shifts (switches) and gradual mood progression (escalations). Each timeline is segmented into subtimelines based on moments of change labels that group consecutive posts with the same label (“In Escalation,” “In Switch,” or “0” for no change). This segmentation, assuming similar mood characteristics within subtimelines, helps identify distinctive features and relationships and was leveraged for structured timeline encoding during modeling.

### Task Formulation

The purpose of these summaries is to extract clinically relevant information from a sequence of potentially lengthy and unfocused social media posts. The goal was to generate a concise summary that can be used by clinicians for a variety of diagnostic and therapeutic purposes. Summaries of this type can contribute to the early detection of mental health risks or illnesses, thus enabling timely intervention and support. They can also help clinicians track and assess patients’ ever-changing mental states, particularly during follow-up periods or between therapy sessions.

Specifically, the summaries aim to address three core aspects of mental health:

1. Overall mental state assessment: identification and evaluation of the individual’s general mood, level of functioning, and any indications of potential mental health issues.
2. Intrapersonal and interpersonal patterns: identifying patterns in self-perception, social interactions, and interpersonal relationships.
3. Mental state fluctuations over time: tracking changes in mood, level of functioning, and symptoms across the timeframe covered by the posts.

Multimedia Appendix 1 presents a comprehensive list of clinical topics for each of these core aspects.

### Computational Modeling Approach

**Figure 1:**
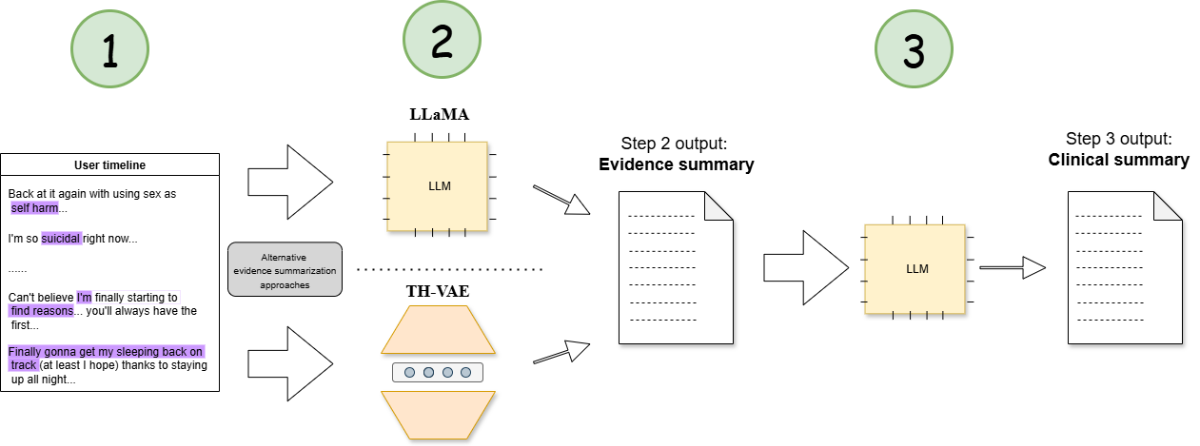
Modeling pipeline for clinical summary generation. LLaMA: large language model Meta AI; LLM: large language model; TH-VAE: timeline-hierarchical variational autoencoder;.

Transforming raw social media timelines into clinically relevant summaries is a complex task that requires intricate reasoning over extended contexts. To address this, we adopt a three-step modeling approach (Figure 1): (1) key phrase identification, (2) first-person timeline summarization (evidence summary), and (3) high-level clinical narrative generation. First, we automatically identify key phrases of potential clinical significance within each segment of the timeline, where a segment is denoted by a moment of change. Next, a first-person abstractive “evidence summary” is generated, guided by the key phrases to distill clinically relevant information. Finally, we generate an abstractive clinical narrative that integrates this evidence summary to offer insights into the user’s mental state.

Figure 1 shows the 3-step modeling pipeline for generating clinical summaries from social media timelines on the TalkLife mental health support platform (2018-2021). Step 1 uses the large language model Meta AI (LLaMA 2; 13B) to highlight clinically relevant phrases. Step 2 generates evidence summaries via either zero-shot LLaMA 2 or the previously published timeline-hierarchical variational autoencoder (TH-VAE) model. Step 3 uses instruction-tuned LLaMA 2 to produce structured, clinically meaningful summaries.

In several places throughout this work, we leverage the in-context learning capability of LLMs. This refers to LLMs’ ability to adapt to new tasks based on examples provided within a prompt by harnessing their language understanding and pattern recognition abilities to generate custom outputs [5,36]. Specifically, we use the in-context learning capability of LLaMA 2 (13B) [37], a popular open-weight LLM, in annotating key phrases, translating the evidence summary into clinical insights, and for one of our baseline methods, in generating the evidence summary. The open-weight nature of LLaMA 2 is crucial for our use case, because it allowed us to use the model without compromising the privacy of sensitive user data.

### Clinical Key Phrase Identification

To set the stage for automatic key phrase identification, 3 graduate students in clinical psychology were trained to highlight clinically relevant key phrases in a subset of three timelines. We then used LLaMA 2 in a few-shot prompt format, primed with these examples, to automatically annotate the remaining timelines.

### Evidence Summary Generation

We explored 2 approaches for generating evidence summaries based on the highlighted clinical key phrases:

#### LLaMA

Leveraging LLaMA 2’s zero-shot capabilities with a prompt instructing it to “write a TLDR of the timeline focusing on the key phrases,” where the key phrases were obtained at the previous step using LLaMA 2. The instruction “TLDR” (ie, Too Long; Didn’t Read), which is a common internet abbreviation, acts as a natural cue for the language model to produce a concise summary.

#### TH-VAE

We use the TH-VAE model [34], a method for generating abstractive summaries of social media timelines, to create evidence summaries that focus on clinically relevant content. This approach is particularly well-suited for addressing the challenges of lengthy and complex timelines, as it incorporates a hierarchical structure that captures both fine-grained details and overarching themes.

The TH-VAE model begins by dividing the timeline into smaller, more manageable coherent segments based on the Moments of Change annotations. Each segment is then encoded into a numerical representation that captures its essential information, with particular emphasis on the clinically relevant key phrases. These segment representations are then combined to create a holistic representation of the entire timeline.

TH-VAE uses a variational autoencoder to compress and reconstruct timeline segments into concise evidence summaries. Its unique architecture specializes in capturing both local details and overarching themes. For full architectural and training details, see Atkins et al [34].

### Clinical Summary Generation

Following Song et al [34], we leverage an instruction-tuned LLM (LLaMA-2-13B-CHAT) to transform the timeline summaries into high-level clinical narratives. This process implements a 2-stage prompting approach. First, in the “map” stage, the LLM is prompted to extract clinically relevant observations from the evidence summary, focusing on specific topics (Multimedia Appendix 1). Then, in the “reduce” stage, the LLM integrates these observations into a cohesive and concise summary, structured along the three core clinical aspects. The full prompts are provided in Multimedia Appendix 2.

### Human Summaries

To establish a gold-standard benchmark for evaluation, we collected human-expert clinical summaries for 33 timelines (randomly sampled from our 500-timeline dataset). Three clinical psychology graduate students, who are fluent in English, annotated the dataset after 2 training sessions, followed by discussion and guideline refinement with an expert clinical supervisor (DA-S).

The annotators first highlighted textual spans conveying information related to the 3 core mental health aspects mentioned above. Next, the annotators wrote comprehensive summaries, dedicating a paragraph to each core aspect. They were instructed to focus on providing the most essential and clinically relevant information that would be useful for a hypothetical clinician reading the summary. Of these 33 expert summaries, 30 were reserved for summary evaluation, and 3 were used for model development and key phrase extraction examples.

### Evaluation Procedure

We compared the performance of the AI models (described below) against the human expert summarizers across three prime dimensions:

- Salient meaning preservation: retaining essential clinical information while discarding less important details.
- Factual consistency: ensuring the summary accurately reflects the timeline’s content without introducing errors or hallucinations.
- Personalization, or diversity: capturing the individual’s unique voice and experiences, avoiding generic language.

While Song et al [34] used automated metrics to evaluate model performance, this paper focuses on assessing and comparing the strengths and weaknesses of both human- and model-generated summaries via human evaluation. Specifically, salient meaning preservation and factual consistency were directly judged by human clinicians. Personalization (diversity) was evaluated through a combination of qualitative inspection and confirming automatic analysis.

We selected three models for evaluation and comparison to the human summaries:

1. TH-VAE: the 3-step model in which the evidence summary is generated by the hierarchical VAE described above.
2. LLaMA: the 3-step model in which the evidence summary is generated directly by the zero-shot LLaMA.
3. Naive LLaMA: a baseline approach, in which the high-level clinical summary is generated by LLaMA in one pass (omitting the Clinical Key Phrase Highlighting and Evidence Summary Generation stages) given the full timeline (see prompt instructions in Multimedia Appendix 2).

### Human Evaluation of the Summaries

The 3 clinical psychology graduate students who generated the human summaries also served as summary raters for their peers. In a blind rating process, the experts each read a full timeline and then evaluated four summaries: 3 model-generated and one written by one of their peers. The summary was rated for five metrics: factual consistency, meaning preservation/general usefulness, and aspect-specific usefulness for the three core clinical aspects on a 1-5 Likert scale.

The annotators were trained to use the evaluation guidelines and had detailed definitions of the assessment criteria (Multimedia Appendix 3). After a preliminary agreement exercise and further clarification, they rated the summaries of the remaining test timelines.

### Qualitative Analysis of the Summaries

To gain deeper insights into the differences between human- and model-generated timeline summaries, we conducted a qualitative evaluation. Two clinicians (a clinical expert and a graduate student in clinical psychology), who were not involved in the model development or human summary creation, independently read 4 timelines and extracted their essential clinical aspects. They then comparatively assessed the human- and model-generated summaries for each timeline, focusing on meaning preservation, factual consistency, and personalization/psychological depth. This aimed to uncover the specific strengths and limitations of each summary type in capturing clinically meaningful insights from the nuances of self-expression patterns in social media posts.

### Quantitative Assessment of Personalization via Diversity

While the qualitative analyses yielded several valuable insights, a particularly salient observation pertained to the potential lack of personalization in the model-generated summaries. This prompted us to further investigate this issue using computational means.

To quantitatively assess the elusive property of personalization in generated summaries, we devised and computed automatic measures capturing the linguistic diversity within the summaries generated by each system. Although linguistic diversity can be attributed to various factors, such as disfluent language or hallucinated irrelevant content, we hypothesize that in high-quality, factually consistent summaries, higher linguistic diversity would correlate with greater personalization. In contrast, a system that yields summaries that are more similar to each other (though characterizing different individuals) would be weaker at using the potential of rich, diverse language to reflect the unique personality and experience of each individual. In short, we conjecture that low diversity remotely signals a generic, nonpersonalized style. We explored 2 complementary dimensions of linguistic diversity, including semantic diversity and surface diversity.

Semantic Diversity

To measure the extent of diversity in conveyed meaning, we used Sentence Transformers (aka SBERT; Reimers & Gurevych, 2019), pretrained language models that encode text meaning into latent embedding vectors. In natural language processing, the semantic similarity between texts is typically computed using the cosine similarity between their corresponding vectors. To assess the variance within each system’s summaries, we computed the centroid vector (the center of the embedding distribution) and calculated the mean cosine similarity between all summary embeddings and this centroid. Lower mean cosine similarity indicates greater semantic diversity. We used various pretrained SBERT models for a robust assessment.

Surface Diversity

Surface diversity pertains to the variation in words and phrases used across summaries, reflecting differences in style and vocabulary. To quantify this, we used ROUGE scores [38], which are commonly used in summarization evaluation. ROUGE measures n-gram overlap between 2 texts; that is, the percentage of consecutive expressions (composed of 1 word for 1-gram, 2 words for 2-gram, etc) that co-occur in both texts relative to the total number of these expressions.

We defined self-ROUGE as a surface linguistic diversity metric based on pairwise ROUGE scores. Specifically, we calculated the mean pairwise ROUGE scores over all pairs of summaries within a system. Lower self-ROUGE scores signify higher surface diversity, indicating less lexical overlap between summaries. We used a variety of ROUGE metrics to capture different aspects of lexical diversity:

- ROUGE-1: unigram (individual word) overlap, providing insights into the diversity of word choices across summaries.
- ROUGE-2, ROUGE-3: bigram or trigram overlap, providing a measure of phrasal diversity and the variety of local word combinations.
- ROUGE-L: overlap of the longest common subsequence between summaries, capturing similarity in the overall structure and information ordering.
- ROUGE-S2 (Skip-Bigram): overlap of skip-bigrams (pairs of words with a gap of up to k=2 words), offering a broader perspective on phrasal diversity.
- ROUGE-SU2 (Skip-Bigram and Unigram): combining unigram and skip-bigram information for a more comprehensive assessment.

Both semantic and surface diversity can serve as proxies for personalization in summaries. Higher semantic diversity suggests a broader range of interpretations and experiences conveyed, while higher surface diversity indicates greater variation in linguistic expression, potentially reflecting more unique and individually tailored clinical descriptions.

To see if the differences in diversity between human and AI summaries were statistically significant, we use permutation testing. This robust method is particularly helpful when we cannot make distributional assumptions about the data.

Specifically, we conduct permutation tests with paired samples. For each iteration, we randomly shuffle system labels (eg, “Human” or “LLaMA”) within each summary pair, keeping summaries from the same source text together. We calculate the difference in mean self-ROUGE between permuted groups and compare it to the observed difference of the original, real groups. By repeating this 1000 times, we obtain a *P* value corresponding to the proportion of permutations where the absolute observed difference is smaller than the absolute permuted difference. Intuitively, this *P* value indicates the likelihood of observing the actual difference by chance. We also calculate Cohen *d* as a standardized measure of effect size.

### Ethical Considerations

This study analyzed user-generated mental health content from TalkLife, a peer support platform. Ethical oversight was provided by two institutional bodies to cover the distinct phases of data collection and subsequent processing:

- Primary data collection: Initial ethical approval for the collection and analysis of the timeline data was obtained from the Institutional Review Board (IRB) of Queen Mary University of London (QMUL; Ref: BSREC 40/19-20, titled "Creating time sensitive sensors from user-generated language & heterogeneous content"). This framework focused on analyzing social media and peer-support content to address the temporal aspects of user-generated data.
- Annotation and processing: Further ethical approval for the specific annotation of timelines and the generation of human and model-based clinical summaries—the core focus of the current work—was granted by the IRB of Bar-Ilan University (BIU).

A formal data-sharing agreement and project proposal were approved by TalkLife, granting the research team data access under strict privacy guidelines. TalkLife users provide consent for research use of their anonymized data through the platform’s terms of service. The dataset shared with researchers was deidentified by TalkLife before release. Additional measures were taken by the research team to ensure user anonymity, including the removal of user handles, timestamps, and other potentially identifying metadata.

Human annotation was carried out by trained in-house research assistants, who produced summaries of user timelines and rated generated summaries. Annotators signed confidentiality agreements and were informed about the potentially distressing nature of the content. They were encouraged to take breaks as needed and were compensated at a rate of NIS 50 (US $14.3) per hour, exceeding the legal minimum wage in Israel and in accordance with institutional guidelines.

No real user timelines or verbatim user-generated text are presented in this manuscript. The paper does not include any images, excerpts, or figures that could potentially reveal the identity of individual users.

Finally, as this study evaluates the use of LLMs to generate clinical summaries of mental health timelines, we acknowledge the ethical risks posed by these technologies. While LLMs demonstrate potential for capturing behavioral and emotional patterns from user-generated content, they are susceptible to factual inaccuracies, hallucinations, and oversimplified interpretations. Such errors could lead to serious consequences if these models were used in clinical decision-making without oversight. We strongly emphasize that automatically generated summaries are not substitutes for professional mental health evaluations. Any future clinical application of such tools must involve trained practitioners who critically assess model outputs within a broader therapeutic framework. Rigorous validation, ethical safeguards, and continued oversight are essential before these systems can be responsibly integrated into mental health care.

This manuscript was edited with the assistance of Gemini Advanced (Google DeepMind), a LLM from Google AI, powered by the Gemini Pro 1.5 model [39]. Details regarding the terms of service and use are provided in Multimedia Appendix 4.

## Results

### Human Evaluation of Summaries

**Figure 2:**
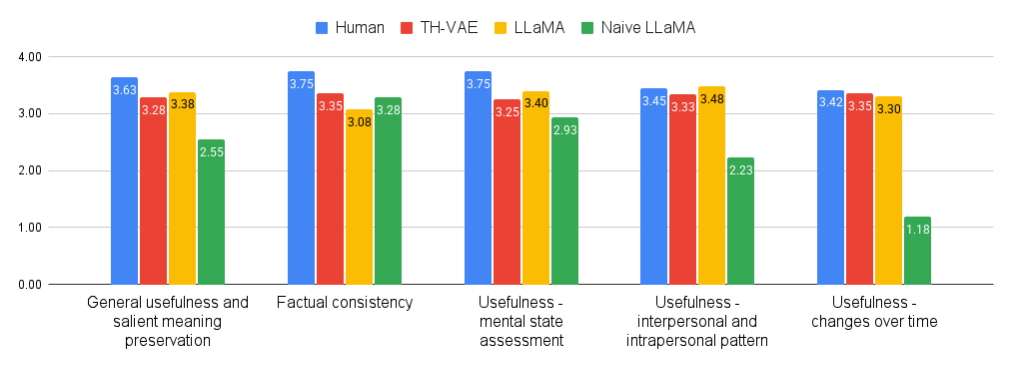
Mean human ratings of timeline summaries. Each metric was rated by a trained clinical psychology graduate student, rating 30 timeline summaries on a 1-5 Likert scale (high is better). LLaMA: large language model Meta AI; TH-VAE: timeline-hierarchical variational autoencoder;.

The results of the quantitative human judgments are summarized in Figure 2.

In terms of general usefulness and salient meaning preservation, the human summaries were rated as significantly more useful than those generated by the naive LLaMA model (*P*<.001; Cohen *d*=1.78). The differences between the human summaries and those generated by TH-VAE (*P*=.07) and LLaMA (*P*=.11) did not reach statistical significance. Both the TH-VAE and the LLaMA summaries, which used a three-step approach and leveraged clinical prompts, were rated significantly higher than the naive LLaMA summaries (*P*<.001; Cohen *d*=1.15 and 1.43, respectively), whereas no significant difference was observed between the former (*P*=.57).

In terms of factual consistency, human-written summaries were rated significantly higher than those generated by TH-VAE (*P*=.003; Cohen *d*=0.92), LLaMA (*P*<.001; Cohen *d*=1.26), or Naive LLaMA (*P*=.003; Cohen *d*=0.82). The comparisons between the modeling approaches, while not statistically significant on this small sample, show that TH-VAE (*P*=.09; Cohen *d*=0.51) and Naive LLaMA (*P*=.23; Cohen *d*=0.31) score higher than LLaMA. LLaMA and TH-VAE performed on par (*P*=.73).

Regarding the specific aspect of usefulness for assessing mental states, the human summaries were rated significantly higher than all the model-generated summaries: LLaMA (*P*=.02; Cohen *d*=0.57), TH-VAE (*P*=.005; Cohen *d*=0.91), and Naive LLaMA (*P*<.001; Cohen *d*=1.34). Naive LLaMA was rated lower than LLaMA (*P*=.001; Cohen *d*=0.69) and TH-VAE (*P*=.06; Cohen *d*=0.51). No significant difference was found between LLaMA and TH-VAE summaries on this dimension (*P*=.40).

In terms of the usefulness of the interpersonal and intrapersonal patterns aspect, the human-written summaries were scored similarly to LLaMA (*P*=.90) and TH-VAE (*P*=.52). No significant difference was found between the LLaMA and the TH-VAE summaries for this dimension (*P*=.33). However, the Naive LLaMA summaries were rated as significantly less useful than all the other systems, including human summaries (*P*<.001; Cohen *d* ranging from 1.65 to 1.95).

Finally, for the aspect of changes in mental state over time, the Naive LLaMA summaries were rated as significantly less useful than all the other systems, including human summaries (*P*<.001; all Cohen *d* measures were greater than 3.4). Aside from this, the permutation tests did not indicate any other statistically significant differences (all *P* values greater than .60).

### Qualitative Analysis of the Summaries

Our qualitative evaluation aimed to provide a deeper characterization of human- vs model-generated summaries. The clinical expertise of the evaluators, coupled with the open-ended nature of this analysis, allowed for insights beyond quantitative metrics.

The evaluators observed a trade-off between comprehensiveness and precision. Model-generated summaries were generally more comprehensive, capturing a wider range of details, while the human summaries were found to be better at focusing on the most clinically salient themes. This suggests that models, though adept at information extraction, may struggle with prioritizing information in line with clinical needs.

Importantly, the human summaries excelled in capturing the nuances of psychological dynamics, thus demonstrating a more personalized and accurate understanding of underlying patterns compared to the models. For example, in one case, the model’s description of a central interpersonal relationship was somewhat generic, whereas the human summary accurately portrayed the related interpersonal pattern, including the causal links between the individual’s desires (wish for closeness), perception of the other (hurtful; due to a prior experience of hurt), reaction toward the other (anger and aggression), and self-reaction (self-harm and despair). This highlights the challenge for models to understand the deeper psychological motivations and interconnectedness of mental elements, even when identifying key details.

On the other hand, the models tended to identify clinically relevant features that the human summaries missed, pointing to their potential to surface subtle patterns or trends at scale. For example, the model noted an individual’s use of aggressive communication and instances of adaptive self-states, both of which could be crucial when tailoring therapeutic interventions. The evaluators noted that the human summarizers were more selective in their choice of content, potentially reflecting a prioritization of the most salient aspects, but possibly also due to inherent human limitations such as fatigue or time constraints.

In terms of the model comparisons, LLaMA and TH-VAE performed comparably, in that both effectively summarized interpersonal/intrapersonal patterns and changes over time but occasionally missed important nuances or made unsupported diagnostic inferences (Mental State Assessment). The basic-prompt model (Naive LLaMA) provided a less comprehensive overview, omitting crucial clinical concepts and failing to reflect significant clinical insights.

Overall, the qualitative evaluation suggests that both human and model-generated summaries have their own unique strengths and limitations. Models offer comprehensiveness and a potential to uncover hidden patterns but may lack the in-depth understanding of human relationships and internal psychological processes that clinicians bring to their work. Human summaries, while more focused, individualized, and clinically insightful, may be limited in their scope and susceptible to human biases.

### Quantitative Assessment of Personalization via Diversity

The results of the semantic diversity analysis, as measured by the mean cosine similarity of sentence embeddings to the centroid, are presented in Table 1. The Naive LLaMA model exhibited the highest variance (ie, lowest intersimilarity) in summary content. Human-generated summaries followed, with the two more performant, three-step models, TH-VAE and LLaMA, demonstrating the least diversity.

**Table 1:** Semantic linguistic diversity.

Sentence embedding model	Naïve LLaMA^a^	TH-VAE^b^	LLaMA	Human
All-mpnet-base-v2	0.79	0.88	0.89	0.85
All-MiniLM-L6-v2	0.77	0.87	0.87	0.82
Paraphrase-albert-small-v2	0.80	0.83	0.84	0.80
Average-word-embeddings-glove.6B.300d	0.95	0.98	0.98	0.95
Average	0.83	0.89	0.89	0.86

^a^LLaMA: large language model Meta AI.

^b^TH-VAE: timeline-hierarchical variational autoencoder.

The difference in diversity between Naive LLaMA and the human summaries was statistically significant for the first 2 sentence embedding models (*P*<.001; Cohen *d* average of 0.84), but not for the other embedding models. In addition, the differences between the human summaries and those generated by the other models (TH-VAE and LLaMA) were all statistically significant (average of *P*=.01 for the 8 comparisons). The Cohen *d* values for these comparisons ranged from 0.60 to 2.40, with an average of 1.19, indicating medium to very large effect sizes depending on the specific embedding model used.

In Table 1 semantic diversity is measured by the mean cosine similarity of sentence embeddings to the centroid embedding. Lower figures indicate lower semantic similarity between summaries of the system, that is, greater diversity.

In terms of surface diversity, Table 2 shows the computed self-ROUGE measures for all systems. The human summaries demonstrated the highest surface diversity across all ROUGE measures. Naive LLaMA came second, whereas the TH-VAE and LLaMA models exhibit the lowest diversity.

The differences between Human and Naive LLaMA were all statistically significant (*P*<.001) except for the ROUGE-L measure. The Cohen *d* values for these significant effects ranged from 1.24 to 1.39, with an average of 1.31, indicating an overall high effect size.

In Table 2 surface diversity is measured by Self-ROUGE scores for each summarization system, calculated using various n-gram overlap metrics. Lower scores indicate lower word overlap between summaries of the system; that is, greater diversity. ROUGE-1 measures unigram (single word) overlap. ROUGE-2 measures bigram (2-word sequence) overlap, ROUGE-3 measures trigram (3-word sequence) overlap, ROUGE-L measures the longest common subsequence overlap, ROUGE-S2 measures skip-bigram overlap (allowing for gaps between the 2 words in the sequence), and ROUGE-SU2 measures skip-bigram plus unigram overlap.

**Table 2:** Surface linguistic diversity.

N-gram overlap measure	Naïve LLaMA^a^	TH-VAE^b^	LLaMA	Human
ROUGE-1	0.36	0.47	0.48	0.28
ROUGE-2	0.10	0.17	0.17	0.05
ROUGE-3	0.04	0.08	0.07	0.01
ROUGE-L	0.23	0.38	0.38	0.22
ROUGE-S2	0.09	0.15	0.15	0.04
ROUGE-SU2	0.16	0.23	0.23	0.11
Average	0.17	0.25	0.25	0.12

^a^LLaMA: large language model Meta AI.

^b^TH-VAE: timeline-hierarchical variational autoencoder.

## Discussion

### Overview

In this study, we explored the potential of LLMs to generate clinically meaningful summaries from social media timelines. We followed both straightforward prompting techniques and state-of-the-art, hybrid architecture to use the power of LLMs for generating such summaries. We then conducted a comprehensive evaluation to assess their performance compared to human summarizers across 3 critical dimensions, including salient meaning preservation, factual consistency, and personalization.

### Salient Meaning Preservation

Capturing clinically relevant information from noisy social media timelines is challenging, demanding both linguistic and clinical understanding. Our evaluation showed that while LLMs can identify key themes, they may struggle to prioritize clinical relevance and discern subtle nuances. The 3-step models, which used key phrase highlighting and clinical prompts, significantly improved performance, closing most of the gap between the naive prompting baseline and human summaries.

The qualitative analysis indicated that even advanced models may favor comprehensiveness over focused clinical insights, which echoes findings in general natural language processing summarization [40]. The inherent complexity of extracting and synthesizing a psychologically coherent narrative from the multifaceted nature of social media posts further amplifies this prioritization hurdle. The human summaries, though less comprehensive, effectively captured the most essential topics from a clinical perspective.

Encouragingly, the gap in scores between the human and the stronger model summaries was small (~0.3 on a 1-5 Likert scale). Future computational research could explore controllable summarization, trading off comprehensiveness and focus based on user needs. From a practical clinical perspective, integrating human and model strengths through collaborative approaches may yield optimal summarization and monitoring outcomes.

### Factual Consistency

Accurate reflection of timeline information is essential for clinical applications. While prior research has examined factual consistency (or faithfulness) in other domains such as news and science [41,42], its evaluation in clinical summaries is less often explored. Our findings reveal that although LLMs can produce largely consistent summaries (average scores exceeding 3 on a 5-point Likert scale), the human summaries were rated significantly higher, particularly when it came to psychological formulations rather than factual details. This suggests that applying abstract clinical concepts, which necessitate a profound understanding of human psychology, to lengthy and intricate first-person narratives poses a significant challenge to current LLM capabilities. Of all the models, TH-VAE was substantially more faithful than LLaMA, highlighting the importance of robust evidence summary generation.

The substantial gap between human and model performance (0.40-0.67 Likert points, large effect sizes) raises questions as to whether future LLMs can overcome these limitations or whether this task reveals the inherent limits of unsupervised language modeling. These results underscore the need for careful scrutiny of LLM-generated summaries in high-stakes clinical settings. Future research should prioritize enhancing clinical accuracy, potentially by incorporating external knowledge bases or grounding inferences on explicit definitions.

### Personalization and Diversity

Personalization, the ability to tailor outputs to an individual’s unique characteristics and needs, is crucial for effective human-computer interaction [43], especially in mental health, where empathy and understanding are paramount [44,45]. Research has explored methods for incorporating personalization into natural language generation [46-48], but achieving true personalization in clinically relevant summaries from social media remains complex.

Our qualitative evaluation highlighted the disparity in personalization between human- and model-generated summaries, where the latter often exhibited a more generic style. This is consistent with the observation that human summarizers demonstrate superior accuracy in capturing psychological insights. The ability to delve deeply into underlying mental mechanisms with high levels of detail facilitates idiosyncratic descriptions, whereas the lack of this type of incisive understanding leads to more generic and obscure narratives. In other words, when it comes to clinically meaningful summaries, accuracy and personalization are densely intertwined.

Quantitatively, we used diversity metrics as a proxy for personalization. Our findings confirmed that the human summaries exhibited significantly greater linguistic diversity, suggesting higher personalization. We explored two complementary linguistic dimensions: content diversity and surface diversity. Content diversity, as assessed through cosine similarity of sentence embeddings, relies on opaque embedding models, thus making it a less interpretable evaluation measure. As shown, while some trends emerged, the comparative picture varied depending on the specific embedding model, leading to inconsistent results.

By contrast, surface diversity, as measured by n-gram overlap, consistently showed that the human summaries were significantly more diverse across all metrics. This suggests that despite their capacity to produce factually accurate and clinically relevant summaries, current models struggle to capture individuals’ unique clinical portraits. We attribute this deficiency to 2 primary factors.

The first is the models’ limited psychological depth, as evidenced by their lower accuracy in capturing more fine-grained psychological formulations as measured in Factual Consistency, which hinders their ability to generate truly personalized summaries. This lack of sharp and clear understanding, required for producing genuinely idiosyncratic, faithful summaries reflecting an individual’s unique experiences, compels them to resort to more generic, repetitive descriptions. Second, the inherent nature of LLMs, shaped by their training data and objectives, may contribute to this tendency toward generic language. Language modeling prioritizes the identification of the most probable text sequence in a given context, which inevitably steers models toward commonplace, frequent linguistic patterns while avoiding idiosyncrasies. This phenomenon is akin to a “regression to the mean” effect, where the model’s predicted text, aiming for maximum probability, defaults to a generic style and repeats “safe” phrases to remain within the realm of high-probability language.

Overall, our findings underscore the difficulties associated with achieving true personalization in LLM-generated clinical summaries of social media timelines. While models demonstrate proficiency in capturing factual information and conveying general clinical impressions, they at the time of this writing fall short of reflecting individuals’ unique voices and experiences. Addressing this limitation requires further research into both the technical aspects of language modeling, such as developing methods for incorporating individual-level information and encouraging more diverse or user-specific language generation, and the incorporation of theoretical psychological understanding into the reasoning processes of computational models. Nevertheless, our conclusions must be tempered due to the lack of robust evaluation criteria for personalization in summarization. While our evaluation represents a first step toward devising such metrics, further work is needed to verify their appropriateness.

### Limitations, Future Directions, and Clinical Implications

While this study provides valuable insights into the potential of LLMs for clinical mental health monitoring on social media, it is important to acknowledge its limitations. First, our dataset was extracted from a forum that primarily caters to teenage girls struggling with self-harm and hence represents a limited range of mental health issues and demographics. It is therefore not fully representative of the variety of online behaviors and mental health presentations. Future work should prioritize dataset expansion to ensure generalizability across diverse clinical populations.

Second, while our evaluation encompassed key dimensions, the quality of a clinical summary is multifaceted and should ideally be assessed from various perspectives. Future work could explore additional, complementary evaluation metrics on the usefulness and clinical applicability of timeline summaries.

Third, our evaluation was limited to English-language content, for which current LLMs perform more strongly compared to low-resourced languages. Future research should investigate the effectiveness of LLMs in summarizing social media content in other languages, especially those with distinct cultural norms and expressions of mental health, to ensure equitable access to these tools across diverse populations.

Despite these limitations and future challenges, our findings have major clinical implications. The ability to generate clinically relevant summaries from social media timelines could revolutionize mental health monitoring and support, empowering clinicians with a more comprehensive and continuous understanding of their patients’ experiences. These summaries have the potential to serve as valuable tools for early detection, intervention, and treatment planning, particularly for individuals who may not actively seek help or have limited access to mental health services. Furthermore, high-quality summaries of large public social media channels could provide a rich resource for psychological and societal research by offering both in-depth individual insights and timely population-level trends.

### Conclusion

This study advances the understanding of how LLMs can support mental health monitoring by systematically evaluating their ability to generate clinically relevant summaries from social media timelines. We evaluated the summarization capabilities of LLaMA 2 (13B) across three modeling approaches, including multistep pipelines designed to enhance clinical relevance. Through a detailed evaluation comparing model-generated summaries to those written by trained clinicians, we provide insight into the current strengths and limitations of generative models in this sensitive domain.

While the tested LLM demonstrated some ability to generate factually coherent and clinically relevant summaries, it fell short in capturing the nuanced psychological depth that human experts provide. These limitations are particularly salient in contexts requiring the recognition of subtle emotional shifts, personal meaning, and interpersonal dynamics. Importantly, the performance we observe may not generalize to other model families or sizes, and further testing with more advanced or fine-tuned models is warranted.

Rather than positioning LLMs as replacements for clinical judgment, our results support a more cautious and integrative view: human-in-the-loop approaches—where clinicians interpret, curate, or augment model outputs—may offer the most promising path forward. Such collaborative frameworks could potentially combine the exhaustiveness and scalability of automated summarization with the psychological accuracy and ethical sensitivity of human professionals.

Continued development, evaluation, and interdisciplinary dialogue will be essential to responsibly integrate LLM-based tools into clinical mental health workflows.

## Data Availability

The datasets generated or analyzed during this study are not publicly available due to privacy and ethical considerations. Specifically, the TalkLife dataset used in this study contains user-generated mental health data collected from the TalkLife peer support platform. To protect user confidentiality and comply with data protection policies, the dataset cannot be shared publicly. Researchers interested in accessing the data must apply directly to TalkLife and obtain approval through their data access request process. Researchers who have obtained approval from TalkLife may contact the authors to request access to the annotated data produced in this study, including the human-written summaries and human evaluation ratings.

## Appendix

Multimedia Appendix 1 Clinical Topics for the Summary.

Multimedia Appendix 2 Model Prompts.

Multimedia Appendix 3 Summary Evaluation Guidelines.

Multimedia Appendix 4 TalkLife Data Sharing Agreement.

## Abbreviations

AI: artificial intelligence
LLaMA: large language model Meta AI
LLM: large language model
TH-VAE: timeline-hierarchical variational autoencoder
VAE: variational autoencoder
