# Supplementary material for: Clinical Summaries of Social Media Timelines for Mental Health Monitoring: Human Versus Large Language Model Comparative Evaluation Study
Source: JMIR Form Res. 2026 Mar 27;10:e71230. doi: 10.2196/71230 (PMC13069367; doi:10.2196/71230)
Supplement: Multimedia Appendix 1 [file formative_v10i1e71230_app1.doc]

## Appendix A - clinical topics for the summary

We crafted a comprehensive list of clinical topics, integrating multiple psychological theories. This list, presented in Table A1, was used for training the human summarizers and for prompting the language models. The task was to capture and summarize information concerning each of these aspects and concepts from user timelines.

## Table A1

### Clinical Aspects and Topics

| **Mental State Assessment** |
| --- |
| Presenting issues (what bothers the person and causes distress; triggers). |
| Mental health symptoms, level of functioning, well-being. |
| Physical symptoms. |
| Risk assessment (previous suicidal attempts, intent to suicide, access to lethal means; hopelessness, social isolation, recent loss, impulsivity, dramatic mood swings). |
| Motivation to change. |
| Lifestyle (diet, physical activity, sleep, alcohol/drug/tobacco use, occupation, environment, screen time, healthcare practices). |
| Agency, coping mechanisms, strengths and resources (what helps the person, how they typically cope with stress and difficulties, resilience). |
| Meaning/goals/direction in life. |
| Behaviour (adaptive and maladaptive behavioral patterns). |
| Important events (present and past events in life; traumatic events). |
| **Intrapersonal and Interpersonal patterns** |
| Main need/wish/desire. |
| Interpersonal relationships (repetitive interpersonal pattern; conflicts; how others are perceived; social support). |
| Self perception, self esteem. |
| **Changes Over Time - Mental State Fluctuations** |
| Emotion (sad, happy, etc). |
| Arousal level (high/low). |
| Emotion regulation strategies. |
| Switches (drastic change of one’s mood). |
| Escalations (intensification in one’s mood). |
| Self understanding (insights about the self and the relationship; ability to reflect and understand repetitive patterns). |
