# Supplementary material for: Clinical Summaries of Social Media Timelines for Mental Health Monitoring: Human Versus Large Language Model Comparative Evaluation Study
Source: JMIR Form Res. 2026 Mar 27;10:e71230. doi: 10.2196/71230 (PMC13069367; doi:10.2196/71230)
Supplement: Multimedia Appendix 2 [file formative_v10i1e71230_app2.doc]

## Appendix B - model prompts

*B.1 Keyphrase Extraction*

Task: Choose key phrases in the following posts.

Text: {example post 1}

Keyphrases:{expert key phrases 1}

Text: {example post 2}

Keyphrases:{expert key phrases 2}

Text: {concatenated posts to annotate}

Keyphrases:

*B.2 Timeline Summarization: LLaMA*

Write a TLDR as the user (first-person), focusing on the keyphrases.

Keyphrases: {extracted keyphrases}

{concatenated posts to summarize}

TLDR:

*B.3 High-level only: Naive LLaMA Baseline*

You are a helpful assistant to an expert therapist who reads social media chronological text written by an individual who has mental health concerns. Summarize the texts below:

{timeline chunk concatenated}

*B.4 Map Prompt: Diagnosis*

Your goal is to describe the individual’s mental state and identify potential indicators that may suggest a mental health diagnosis, considering the following aspects:

1. Presenting Issues: What are the main concerns or stressors evident in the individual's posts? What triggers seem to affect their mental state?

2. Mental Health Symptoms and Functioning: Does the individual exhibit any mental health symptoms? How are their mood, energy levels, and interest in usual activities? Are there noticeable changes in sleep patterns, appetite, concentration, or social interactions? How do they describe their overall wellbeing and functioning in daily activities?

3. Mental Health Treatment History: Has the individual been in contact with mental health professionals such as psychiatrists or psychotherapists? Are there mentions of current or past outpatient or inpatient mental health treatments? Do they reference taking psychiatric medications?

4. Physical Health: Are there any current or past physical health issues, medical conditions, hospitalizations, or surgeries mentioned?

5. Risk Assessment: Is there evidence of previous suicidal attempts or current suicidal thoughts? Do they have access to lethal means? What level of hopelessness is expressed? Do they discuss social isolation, recent losses, impulsivity, or dramatic mood swings?

6. Lifestyle Factors: What do the individual’s posts reveal about their lifestyle habits, such as diet, physical activity, sleep patterns, occupation, environment, screen time, and healthcare practices?

7. Substance Use: Are there any references by the individual to the use of substances like alcohol, drugs, or tobacco? If so, how frequently do they use these substances?

8. Significant Life Events and Family History: Are there references to significant life events like divorce, loss of a close person, experiences of abuse, or neglect? Is there any mention of psychiatric problems or treatments among family members?

9. Motivation and Coping Strategies: What does the individual express about their motivation for change? How do they cope with stress and difficulties? What strengths and resources do they have? What seems to help them? How resilient do they appear? Do they discuss having direction, meaning, or goals in their life?

You must not make anything up. Keep the description concise and only describe observations if they are fully supported by the text.

Here are the texts:

{Timeline summary}

*B.5 Map Prompt: Intrapersonal and Interpersonal Patterns*

Your goal is to identify the person's main intrapersonal and interpersonal pattern, considering the following aspects:

1. Wish/Need/desire/intention/expectation: What is the person’s most dominant need, desire, intention, expectation from others and from themselves? Are there any other needs or wishes that might be indicated in a less obvious way? How well does the individual communicate their needs/ wishes with others?

2. Response of Others: How does this person typically perceive the emotions, behaviors, and thoughts of others? Are there any other perceptions of the other that might be indicated in a less obvious way? Is the individual capable of acknowledging the complex nature of others?

3. Response of self to others: How does this person tend to feel and react to others? Are there any other reactions towards others that might be indicated in a less obvious way?

4. Response of self to self: What is the individual’s most dominant emotion, behavior and cognition toward oneself? Are there any other emotions and cognitions towards the self that might be indicated in a less obvious way? What is the level of self-compassion and acceptance of strengths and vulnerabilities?

5. Patterns: What is this individual’s predominant dysfunctional intrapersonal and interpersonal pattern? What is this individual’s predominant adaptive intrapersonal and interpersonal pattern?

You must not make anything up. Keep the description concise and only describe observations if they are fully supported by the text.

Here are the texts:

{Timeline summary}

*B.6 Map Prompt: Changes over Time*

Your goal is to understand and summarize changes over time in this individual’s mood, well-being and functioning (individual/self well being; interpersonal well being including family and close relationships; social well-being, including work, school and friends). From the following text, identify whether there are changes in the individual's expressed mood, well-being, and functioning.

a. Overall, does the mood/ well being/ functioning stay consistent or fluctuate over time?

b. Are there specific positive to negative changes? Which events (personal or interpersonal) are associated with these changes?

c. Are there specific negative to positive changes? Which events (personal or interpersonal) are associated with these changes?

Answer each concisely and reply None if not available. You must not make anything up. Keep the description concise and only describe observations if they are fully supported by the text.

Here are the texts:

{Timeline summary}

*B.7 Reduce Prompt*

Rewrite these sentences about the individual so that they form a single clear and coherent document. Combine redundant sentences, but do not remove any clinical information or information about interpersonal relationships. You must not make anything up.

{Previously generated summaries}
