# Supplementary material for: Clinical Summaries of Social Media Timelines for Mental Health Monitoring: Human Versus Large Language Model Comparative Evaluation Study
Source: JMIR Form Res. 2026 Mar 27;10:e71230. doi: 10.2196/71230 (PMC13069367; doi:10.2196/71230)
Supplement: Multimedia Appendix 3 [file formative_v10i1e71230_app3.doc]

## Appendix C. Summary Evaluation Guidelines

You will be asked to assign each summary a score from 1 (worst) to 5 (best) based on the criteria below. Please read each summary carefully. For each aspect, please carefully consider how you would score that summary, independent of other aspects and your scores for other summaries.

## Table C1

### Summary Evaluation Metrics

| Factual consistency | A factually consistent summary accurately reflects the content of the timeline. It does not contain information that is not present in the timeline. |
| --- | --- |
| General usefulness and  Salient meaning preservation | A useful summary should help the clinician understand the client’s condition. It should contain the most important information of the timeline. It does not include parts of the timeline that are less important.  In our project, we would like you to focus on **clinically important information**. |
| Usefulness (diagnosis) | The summary provides useful information about the individual's diagnosis (such as presenting issues, mental health & physical symptoms, risk assessment, behaviour). |
| Usefulness (interpersonal and intrapersonal pattern) | The summary provides helpful information about the individuals' main needs and patterns of self and other relationships. |
| Usefulness  (changes over time) | The summary provides useful information about the individual's changes over time in emotion/cognition and behaviour. Where appropriate, it should help connect information between events and the individual’s responses. |

| **Factual Consistency**    A factually consistent summary accurately reflects the content of the timeline. It does not contain information that is not present in the timeline. | - 1 - Not at all factually consistent: The summary contains significant inaccuracies or misrepresentations, completely misaligning with the timeline's content. - 2 - Mostly not factually consistent: The summary contains significant inaccuracies or misrepresentations, poorly reflecting the timeline's content. - 3 - Somewhat factually consistent: The summary is somewhat accurate, with several inaccuracies or omissions, but retains a basic reflection of the timeline’s content. - 4 - Mostly factually consistent: The summary is largely accurate, with minor inaccuracies or omissions that do not majorly distort overall understanding. - 5 - Fully factually consistent: The summary is completely accurate, perfectly aligning with the timeline's content without discrepancies. |
| --- | --- |
| **General Usefulness and Salient Meaning Preservation**    A useful summary should help the clinician understand the client’s condition. It should contain the most important information of the timeline. It does not include parts of the timeline that are less important. | - 1 - Not at all useful: The summary fails to capture any essential information, significantly misrepresenting or omitting critical aspects of the individual’s condition. - 2 - Slightly useful: The summary includes some important details but primarily focuses on irrelevant or less critical information. - 3 - Moderately useful: The summary captures important information but still includes less relevant details or omits minor key elements. - 4 - Very useful: The summary highlights most of the crucial information, with only minor irrelevant details. - 5 - Extremely useful: The summary encapsulates all critical information, providing a comprehensive and clear understanding of the individual’s condition, without providing irrelevant information. |
| **Usefulness (mental state assessment)**    The summary provides useful information about the individual's diagnosis (presenting issues, mental health & physical symptoms, risk assessment, behavior). | - 1 - Not at all useful: The summary fails to provide information regarding the individual's diagnosis, or it clearly distorts the individual’s diagnosis by incorrectly identifying diagnostic elements. - 2 - Slightly useful: The summary provides minimal information related to the individual’s diagnosis. While the summary includes some correct diagnostic elements, it generally contains irrelevant or incorrect details and omissions. - 3 - Moderately useful: The summary is generally accurate about the individual’s diagnosis but only describes the more obvious aspects, with some information possibly missing or unclear. - 4 - Very useful: The summary accurately identifies the individual’s diagnosis and captures almost all the essential diagnostic information with only minor gaps. - 5 - Extremely useful: The summary is comprehensive and accurately details all aspects of the individual's diagnosis. |
| **Usefulness (interpersonal and intrapersonal pattern)**    The summary provides helpful information about the individuals' main needs and patterns of self and other relationships. | - 1 - Not at all useful: The summary provides no insight into the individual's interpersonal and intrapersonal patterns. - 2 - Slightly useful: The summary provides a minimal understanding of interpersonal and intrapersonal patterns. - 3 - Moderately useful: The summary covers some key aspects of the individual's interpersonal and intrapersonal patterns but may lack depth or miss important elements. - 4 - Very useful: The summary provides a comprehensive overview of the individual’s interpersonal and intrapersonal patterns, with only slight gaps or generalizations. - 5 - Extremely useful: The summary gives a detailed and complete understanding of the individual's interpersonal and intrapersonal patterns. |
| **Usefulness**  **(changes over time)**    The summary provides useful information about the individual's changes over time in emotion/cognition and behavior. Where appropriate, it should help connect information between events and the individual’s responses. | - 1 - Not at all useful: The summary fails to provide any accurate information about whether there are changes in the individual over time. - 2 - Slightly useful: The summary includes information about changes, but they are generally inaccurate and overlook key developments/connections, or they generally contain irrelevant information. - 3 - Moderately useful: The summary accurately describes whether there are changes, although there may be some weaknesses or omissions as well as irrelevant information. - 4 - Very useful: The summary accurately describes whether there are changes and where available offers helpful insights. - 5 - Extremely useful: The summary accurately describes whether there are changes and where available provides clear, well-connected insights about the individual’s development over time. |
