# Supplementary material for: Clinical Summaries of Social Media Timelines for Mental Health Monitoring: Human Versus Large Language Model Comparative Evaluation Study
Source: JMIR Form Res. 2026 Mar 27;10:e71230. doi: 10.2196/71230 (PMC13069367; doi:10.2196/71230)
Supplement: Multimedia Appendix 4 [file formative_v10i1e71230_app4.pdf]

# TalkLife Data Sharing Agreement

| | | |
|---|---|---|
| **Parties** | **TALKLIFE LTD** incorporated and registered in England and Wales with company number 09104043 whose registered office is at 40 Huller and Cheese, Redcliff Backs, Bristol, England, BS1 6WJ (**TalkLife**) | |
| | **Nottingham Trent University** of 50 Shakespeare Street, Nottingham, NG1 4FQ (**NTU**) and **The Alan Turing Institute**of British Library, 96 Euston Rd, London, NW1 2DB (**Turing**). | |
| **Terms** | This agreement incorporates TalkLife's Terms and Conditions as appended to this Order Form. | |
| **Description of Data** | TalkLife Dataset. The Dataset is a de-identified database of user profiles, posts, comments, reactions, age, gender, app interactions, user behaviour, mood tracking, timestamps, and triggering content. User interaction data such as how many times a user has viewed another users profile, and what the user then went on to do. | |
| **Project** | Title: Longitudinal User Modelling and Clustering for Mental Health Using Language, Asynchronous and Heterogeneous Data<br><br>Start date (duration): August 2019 (10 years)<br>Project Leads: Dr Maria Liakata (ATI) and Dr Eiman Kanjo (NTU)<br>Proposed team: Adam Tsakalidis (ATI), Bo Wang(ATI/Oxford), Dong Nguyen(ATI/Utrecht), Theo Damoulas(ATI/Warwick), Marya Bazzi (ATI/Oxford), Elena Kochkina(ATI/Warwick), Nicole Peinelt (ATI/Warwick), Terry Lyons (ATI/Oxford), Maria Wolters (ATI/Edinburgh), Rob Procter(ATI/Warwick), Kate Saunders (Oxford), Alanoud Alharbi (PhD student at NTU), PDRA (NTU), Dr. Thanasis Tsanas (Oxford/Edinburgh-Usher Institute of Population Health Sciences and Informatics), Dr. Donald MacIntyre (Centre for Clinical Brain Sciences, Division of Psychiatry, University of Edinburgh).<br><br>Background and Motivation: Monitoring and assessing mental health through smart devices and social media is an active and promising area of research [1-8]. A lot of work in this area aims to distinguish between users of different types (e.g. users with a condition vs controls) based on their social media output [2-4,6]. Another line of work aims to use data derived from a group of users (e.g. data from mobile phone sensors) in order to predict a target value such as mood or stress scores based on a psychometric test or discrete labels [1,5,7].<br>In previous work, performance evaluation is usually conducted using cross-fold validation, which gives over-optimistic results and poses important questions with respect to the applicability of such systems in real world applications [8]. Moreover, medical researchers are- justifiably so- suspicious of any models that may tend to overfit or lack interpretability, with the caveat of opting for simple correlations or at best linear models which are limited in their predictive power.<br><br>Objectives and Methods: We aim at working into two parallel directions that share in common the longitudinal aspect of user modelling as a means towards assessing mental well-being. In particular, our project is divided into creating (a) robust longitudinal NLP models for capturing changes in language use and other online behaviour over time (an objective to be undertaken by the ATI team) and (b) communities of users that share common online behavioural patterns (to be undertaken by the NTU team), both as proxies for assessing mental well-being. Both (a) and (b) involve:<br>Longitudinal user modelling, either on a personalised (a) or on a community basis (b).<br>Developing methods for measuring the similarities of users across different points in time, with a primary focus on latent asynchronous linguistic and heterogeneous (a) or online behavioural (b) features. | |

| | |
|---|---|
| | Sharing these common modelling aspects, the two aims will be approached in a distinct manner, with different methodologies. In particular, for objective (a) the ATI team plans to:<br>(a1) Build latent user representations to identify normal states and anomalies for individuals by considering changes in their language use and online behaviour over time. Language use will be defined in terms of vocabulary, syntactic patterns, as well as local/global coherence.<br>(a2) Identify similarities between users at different points in time to be able to characterise as quickly as possible the development of new users for whom we have no history.<br>(a3) Use the above to help train both intra-and inter-user predictors of mood change, on a personalised manner, tackling the issues presented in [8].<br>(a4) Provide interpretable summaries of these findings that can be used by health experts and individuals and work on possible medical interventions together with clinical experts.<br>ATI plans to explore a number of different supervised (multiple kernel learning, recurrent neural networks) and unsupervised methods (multi-layer networks) suited to modelling sequential data as well as different ways of representing user information. For example, there are important decisions to be made in terms of inferring missing data or dealing with data sparsity. A key aspect of the modelling involves building latent and temporally sensitive representations that combine textual, static (e.g., demographic) and asynchronous information (e.g., network) that can represent a user or a feature in a longitudinal fashion and study their effectiveness into capturing mental well-being (either in an intra- or in an inter-user validation setting).<br><br>For objective (b), the NTU team plans to:<br>(b1) Extract various features and mental health states based on users' interests, lifestyle, app interaction and communication with others (exploratory phase)<br>(b2) Apply methods to group users into communities/groups based on their interests, behaviour and inter-relationship with their mental health.<br>(b3) Identify and characterise the main factors contributing to the mental wellbeing of users and the relationship between these variables and triggers (e.g. impact of sleep on depression and vice versa) based on keywords, time of interaction etc.<br>(b4) Explore the potential of Deep Learning in studying the temporal dynamics architecture and generating automated messages to support users.<br>(b5) Look at a variety of big data algorithms and techniques, including storage, encryption, visualisation, optimisation, compression.<br>NTU plans to apply descriptive and inferential statistical techniques as well as graph theory, factorial analysis and clustering algorithms to group users into communities. This type of analysis will allow us to understand the relationships between mental health problems, triggers and consequences (including peer influence impact of relationships, parents, college, etc).<br><br>References<br>[1] Canzian, L. and Musolesi, M., 2015, September. Trajectories of depression: unobtrusive monitoring of depressive states by means of smartphone mobility traces analysis. In Proceedings of the 2015 ACM international joint conference on pervasive and ubiquitous computing (pp. 1293-1304). ACM.<br><br>[2] Coppersmith, G., Dredze, M., Harman, C., Hollingshead, K. and Mitchell, M., 2015. CLPsych 2015 shared task: Depression and PTSD on Twitter. In Proceedings of the 2nd Workshop on Computational Linguistics and Clinical Psychology: From Linguistic Signal to Clinical Reality (pp. 31-39).<br><br>[3] De Choudhury, M., Gamon, M., Counts, S. and Horvitz, E., 2013. Predicting depression via social media. ICWSM, 13, pp.1-10.<br><br>[4] De Choudhury, M., Kiciman, E., Dredze, M., Coppersmith, G. and Kumar, M., 2016, May. Discovering shifts to suicidal ideation from mental health content in social media. In Proceedings of the 2016 CHI conference on human factors in computing systems (pp. 2098-2110). ACM.<br><br>[5] LiKamWa, R., Liu, Y., Lane, N.D. and Zhong, L., 2013, June. Moodscope: Building a mood sensor from smartphone usage patterns. In Proceeding of the 11th annual international conference on Mobile systems, applications, and services (pp. 389-402). ACM.<br><br>[6] Milne, D.N., Pink, G., Hachey, B. and Calvo, R.A., 2016. Clpsych 2016 shared task: Triaging content in online peer-support forums. In Proceedings of the Third Workshop on Computational Linguistics and Clinical Psychology (pp. 118-127).<br><br>[7] Tsakalidis, A., Liakata, M., Damoulas, T., Jellinek, B., Guo, W. and Cristea, A., 2016. Combining heterogeneous user generated data to sense well-being. In Proceedings of COLING 2016, the 26th International Conference on Computational Linguistics: Technical Papers (pp. 3007-3018). |

| | |
|---|---|
| | [8] Tsakalidis, A., Liakata, M., Damoulas, T. and Cristea, A.I., 2018. Can We Assess Mental Health through Social Media and Smart Devices? Addressing Bias in Methodology and Evaluation. arXiv preprint arXiv:1807.07351. |
| **Licence Fee** | £8,500 + VAT (Fee payable by The Alan Turing Institute) |
| **Address for Notices** | **TalkLife:**<br>**TalkLife Limited,**<br>1 Victoria Street<br>Bristol<br>BS1 6AA<br><br>**NTU:**<br>**Nottingham Trent University** of 50 Shakespeare Street, Nottingham, NG1 4FQ<br><br>**Turing**:<br>**The Alan Turing Institute** of British Library, 96 Euston Rd, London, NW1 2DB |
| **Other as applicable** | **Clause 10 – Data Protection** |
| **THIS AGREEMENT** has been executed by or on behalf of the parties on the date below. | |
| **Signed by a duly authorised signatory of TalkLife Ltd** | ____ Signature<br>28/08/2019 Date<br>Jamie Druitt Name<br>CEO Status<br>**TalkLife Limited** |
| **Signed by a duly authorised signatory of**: | ____ Signature<br>28/8/19 Date<br>Barbara Pierscionek Name<br>Associate Dean for Research Status<br>**Nottingham Trent University**<br><br>____ Signature<br>23/08/2019 Date<br>Adrian Smith Name<br>Institute Director and Cheif Executive Status<br>**The Alan Turing Institute** |

**TERMS AND CONDITIONS**

1 **INTERPRETATION**

1.1 The definitions and rules of interpretation in this clause apply in this Agreement.

**Agreement:** the Order Form together with these terms and conditions.

**Confidential Information**: all confidential information (however recorded or preserved) disclosed by a party or its employees, officers, representatives, advisers or sub-contractors involved in the provision or receipt of the Data who need to know the confidential information in question (**Representatives**) to the other party and that party's Representatives in connection with this Agreement, which is either labelled as such or else which should reasonably be considered as confidential because of its nature and the manner of its disclosure.

**Customer:** NTU, the Turing or both as interpreted in accordance with clause 1.14.

**Customer System:** any information technology system or systems owned or operated by the Customer from which Data is received in accordance with this Agreement.

**Customer User:** any employee, worker, student or sub-contractor of the Customer authorised by the Customer to work on the Project including but not limited to the individuals mentioned under the Project.

**Customer User Restrictions:** the obligations on the Customer set out in clause 6.

**Data:** the data licensed by TalkLife to the Customer, as described in the Order Form.

**Effective Date:** the date the Order Form is signed by all parties.

**Intellectual Property Rights:** all patents, rights to inventions, utility models, copyright and related rights, trade marks, service marks, trade, business and domain names, rights in trade dress or get-up, rights in goodwill or to sue for passing off, unfair competition rights, rights in designs, rights in computer software, database rights, semi-conductor topography rights, moral rights, rights in confidential information (including know-how and trade secrets) and any other intellectual property rights, in each case whether registered or unregistered and including all applications for and renewals or extensions of such rights, and all similar or equivalent rights or forms of protection in any part of the world.

**Licence Fee:** the licence fee payable by the Customer set out in the Order Form and in accordance with clause 4.

1.2 Clause and paragraph headings shall not affect the interpretation of this Agreement.

1.3 A **person** includes a natural person, corporate or unincorporated body (whether or not having separate legal personality).

1.4 The Order Form forms part of this Agreement and shall have effect as if set out in full in the body of this Agreement. Any reference to this Agreement includes the Order Form.

1.5 A reference to a **company** shall include any company, corporation or other body corporate, wherever and however incorporated or established.

1.6 Unless the context otherwise requires, words in the singular shall include the plural and in the plural shall include the singular.

1.7 Unless the context otherwise requires, a reference to one gender shall include a reference to the other genders.

1.8 A reference to a statute or statutory provision is a reference to it as amended, extended or re-enacted from time to time.

1.9 A reference to a statute or statutory provision shall include all subordinate legislation made from time to time under that statute or statutory provision.

1.10 Unless otherwise expressly stated in this Agreement, a reference to **writing** or **written** includes email.

1.11 References to clauses are to the clauses of this Agreement.

1.12 Any words following the terms **including**, **include**, **in particular** or **for example** or any similar phrase shall be construed as illustrative and shall not limit the generality of the related general words.

1.13 The terms of this Agreement shall prevail over and to the exclusion of any other terms that the Customer seeks to impose or incorporate, or which are implied by trade, custom, practice or course of dealing.

1.14 To reflect the intention that TalkLife will provide separate copies of the Data to each of NTU and the Turing and that each of NTU and Turing will be responsible for ensuring compliance with this Agreement only in respect of the Data provided to them by TalkLife, the following shall apply to the interpretation of this Agreement:

1.14.1 the licence granted in clause 2 shall apply separately to each of NTU and the Turing in respect of the

Data provided to them;

1.14.2 the rights to receive a copy of the Data in clause 3 shall apply separately to each of the Turing and NTU;

1.14.3 the payment obligations in clause 4 shall apply only to the Turing, save that NTU's right to receive and make use of any Data is subject to the Turing's compliance with these obligations;

1.14.4 the audit obligations in clause 5 shall only apply to each of NTU and the Turing only in respect of their compliance with their own obligations under this Agreement;

1.14.5 each of NTU and the Turing shall be responsible for complying with the Customer User Restrictions in clause 6 only as they relate to the Data provided to them individually by TalkLife and only to any Customer User's use of such Data;

1.14.6 the unauthorised use provisions in clause 7 shall apply to each of NTU and the Turing only in respect of their own acts or defaults (for the avoidance of doubt, if the termination right under clause 7.1.2 is triggered by either NTU or the Turing, any subsequent termination by TalkLife will apply to the whole Agreement and not just to the party whose act or default triggered the termination);

1.14.7 the provisions of clauses 8, 9, 10, 12, 13, 14, 16 and 18 relating to publication, confidentiality, data protection, intellectual property rights, warranties, limitation of liability, consequences of termination and assignment shall apply separately to each of NTU and the Turing;

1.14.8 the provisions relating to security and passwords in clause 11 will apply separately to each of NTU and the Turing but in each case only in respect of the Data and Confidential Information they receive from TalkLife;

1.14.9 the provisions relating to term and termination in clause 15 shall apply separately as between TalkLife and NTU and as between TalkLife and the Turing (for the avoidance of doubt, if a TalkLife termination right is triggered by either NTU or the Turing, any subsequent termination by TalkLife will apply to the whole Agreement and not just to the party whose act or default triggered the termination);

1.14.10 the provisions of clause 17 relating to force majeure shall apply separately between TalkLife and NTU and between TalkLife and the Turing; and

1.14.11 clauses 19 to 27 shall take their ordinary and natural meaning.

## 2 LICENCE

In consideration of the Licence Fee, TalkLife hereby grants to the Customer a non-exclusive, non-transferable, revocable, worldwide licence for the duration of the Project to use, modify and adapt the Data for the Project only and subject to the Customer User Restrictions.

## 3 DELIVERY

3.1 TalkLife shall provide the Customer on the agreed form of recordable media, or make available for download, one copy of the Data within a reasonable period following the receipt of the Licence Fee by TalkLife from the Customer. Risk in any tangible media on which the Data is delivered shall pass on delivery.

3.2 TalkLife may change at any time, with as much prior notice to the Customer as is reasonably practicable:

3.2.1 the content, format or nature of Data; and

3.2.2 the means of access to the Data.

3.3 Any dates quoted for delivery of the Data are approximate only, and the time of delivery is not of the essence. TalkLife shall not be liable for any delay in delivery of the Data that is caused by an event within the scope of clause 17 or the Customer's failure to provide TalkLife with adequate delivery instructions or any other instructions that are relevant to the supply of the Data.

## 4 LICENCE FEE

4.1 In consideration of the Data, the Customer shall pay to TalkLife the Licence Fee.

4.2 TalkLife shall invoice the Customer on or after the Effective Date for the Licence Fee due for the Data and the Customer shall pay each invoice within 30 days after the date of such invoice.

4.3 Time shall be of the essence regarding the Customer's obligations to make payments in accordance with this clause 4 and breach of such obligations shall be deemed to be a material breach for the purpose of clause 15.2.2.

4.4 TalkLife may charge interest at an annual rate of 4% above the base rate of Metro Bank plc., calculated on a daily basis in respect of any sum which is due and unpaid, that interest to run from the date on which that sum is due and payable until receipt by TalkLife of the full amount, whether before or after judgment.

4.5 The Licence Fee is exclusive of VAT or any other applicable sales tax in the UK or elsewhere, which shall be paid by the Customer at the rate and in the manner for the time being prescribed by law.

## 5 AUDIT

5.1 The Customer shall keep, in paper and electronic form, at its normal place of business detailed, accurate and up-to-date records (**Records**) showing, during the previous three years the steps taken by the Customer to comply with the Customer User Restrictions. The Customer shall ensure that the Records are sufficient to enable TalkLife to verify the Customer's compliance with its obligations under this clause 5.

5.2 The Customer shall permit TalkLife and its third party representatives, on reasonable notice during normal business hours, but without notice in the case of any reasonably suspected breach of clauses 2, 6, 7 or 10, to:

5.2.1 gain (physical and remote electronic) access to, and take copies of, the Records and any other information held at the Customer's premises or on the Customer System; and

5.2.2 inspect all Records and Customer Systems relating to the use, modification, adaptation, permissioning and control of the Data,

for the purpose of auditing the Customer's compliance with its obligations under this Agreement including the Customer User Restrictions and clauses 2, 8, 9 and 10. Such audit rights shall continue for one year after termination of this Agreement. The Customer shall give all necessary assistance to the conduct of such audits during the term of this Agreement and for a period of three years after termination of this Agreement.

## 6 CUSTOMER USER RESTRICTIONS

6.1 The Customer shall:

6.1.1 ensure that only Customer Users have access to and use the Data;

6.1.2 procure that Customer Users comply with the terms of this Agreement and at all times be responsible for such Customer Users' compliance with the terms of this Agreement;

6.1.3 not redistribute, re-disseminate, sublicense or transfer the Data;

6.1.4 unless otherwise expressly agreed by the parties in writing, complete the process set by, and ensure any use, modification or adaptation of Data receives approval from, the relevant Institutional Review Board for human subjects research and/or any other applicable ethics approval;

6.1.5 carry out the Project in accordance with good academic practice and/or good industry practice (as applicable);

6.1.6 not use the Data for any purpose contrary to any law or regulation or any regulatory code, guidance or request;

6.1.7 not extract, reutilise, use, exploit, redistribute, redisseminate, copy or store the Data for any purpose not expressly permitted by this Agreement; and

6.1.8 not do anything which may damage the reputation of TalkLife, the Data or the Data, including by way of using the Data (wholly or in part) in any manner which is pornographic, racist or that incites religious hatred or violence.

## 7 UNAUTHORISED USE

7.1 If any unauthorised use is made of the Data and such use is attributable to the act or default of, or through, the Customer (including breach of any Customer User Restrictions or the scope of the licence at clause 2) then, without prejudice to TalkLife's other rights and remedies:

7.1.1 the Customer shall immediately be liable to pay TalkLife an amount equal to the Licence Fee that TalkLife would have charged, had

TalkLife or the Customer (as the case may be) authorised the unauthorised user at the beginning of the period of that unauthorised use together with interest at the rate provided for in clause 4.4 from the date of that unauthorised use to the date of payment; and

7.1.2 TalkLife may terminate this Agreement with immediate effect on written notice to the Customer.

8 **PUBLICATION**

Notwithstanding the provisions of clause 9, TalkLife recognises that the Customer may wish to publish information derived from the Data in the academic press and/or to divulge such information at academic meetings or symposia which information may include the Data. Any such publication or disclosure may only be made after notifying TalkLife of such publication or disclosure.

9 **CONFIDENTIALITY**

9.1 The term Confidential Information does not include any information that:

9.1.1 is or becomes generally available to the public (other than as a result of its disclosure by the receiving party or its Representatives in breach of this clause 9);

9.1.2 was available to the receiving party on a non-confidential basis before disclosure by the disclosing party;

9.1.3 was, is, or becomes, available to the receiving party on a non-confidential basis from a person who, to the receiving party's knowledge, is not bound by a confidentiality agreement with the disclosing party or otherwise prohibited from disclosing the information to the receiving party;

9.1.4 was known to the receiving party before the information was disclosed to it by the disclosing party; or

9.1.5 the parties agree in writing is not confidential or may be disclosed.

9.2 Each party shall keep the other party's Confidential Information confidential and shall not:

9.2.1 use any Confidential Information except for the purpose of exercising or performing its rights and obligations under this Agreement for the Project; or

9.2.2 disclose any Confidential Information in whole or in part to any third party, except as expressly permitted by this clause.

9.3 A party may disclose the other party's Confidential Information to those of its Representatives who need to know that Confidential Information for the Project, provided that:

9.3.1 it informs those Representatives of the confidential nature of the Confidential Information before disclosure; and

9.3.2 at all times, it is responsible for the Representatives' compliance with the confidentiality obligations set out in this clause 9.

9.4 The Customer acknowledges that TalkLife's Confidential Information includes the Data.

9.5 A party may disclose Confidential Information to the extent required by law, by any governmental or other regulatory authority, or by a court or other authority of competent jurisdiction provided that, to the extent it is legally permitted to do so, it gives the other party as much notice of the disclosure as possible.

9.6 Each party reserves all rights in its Confidential Information. No rights or obligations in respect of a party's Confidential Information, other than those expressly stated in this Agreement, are granted to the other party, or are to be implied from this Agreement.

9.7 The provisions of this clause 9 shall continue to apply after termination of this Agreement.

10 **DATA PROTECTION**

10.1 Each Party acknowledges and agrees that the Parties will share Data under this Agreement and as such TalkLife shall ensure that, prior to providing any Data to the Customer, it has all appropriate legal bases (whether consent or otherwise) necessary to enable lawful transfer of the Data to the Customer. Once such Data has been provided to the Customer, the Parties acknowledge and agree that each Party shall act as separate Data Controllers when Processing such Data and

10.1.1 TalkLife shall be a Data Controller where it is processing Data for the

purpose of its service provision;

10.1.2 the Customer shall be a Data Controller where it is processing Data for the purpose of the Project;

10.1.3 the Parties do not anticipate that either will act as a data processor on behalf of the other Party and neither Party is authorised or instructed to act as a data processor on behalf of each other.; and

10.2 the Parties will not act as joint data controllers in common.

10.3 Each Party shall ensure that it complies with its respective obligations under Data Protection Legislation.

10.4 Each Party shall indemnify and keep indemnified (the "Indemnifying Party" )the other Party (the "Indemnified Party") against all costs, claims, damages, expenses, fines and/or penalties incurred by or imposed on the Indemnified Party due to any failure by the Indemnifying Party to comply with its obligations under Data Protection Legislation

11 **SECURITY AND PASSWORDS**

11.1 The Customer shall ensure that the Data and TalkLife's Confidential Information is kept secure and in an encrypted form, and shall use the best available security practices and systems applicable to the use of the Data and TalkLife's Confidential Information to prevent, and take prompt and proper remedial action against, unauthorised access, copying, modification, storage, reproduction, display or distribution of the Data and TalkLife's Confidential Information.

11.2 If the Customer becomes aware of any misuse of any Data and/or TalkLife's Confidential Information, or any security breach in connection with this Agreement that could compromise the security or integrity of the Data and/or TalkLife's Confidential Information or otherwise adversely affect TalkLife, the Customer shall, at the Customer's expense, promptly notify TalkLife and fully co-operate with TalkLife to remedy the issue as soon as reasonably practicable.

11.3 The Customer agrees to co-operate with TalkLife's reasonable security investigations.

12 **INTELLECTUAL PROPERTY RIGHTS**

12.1 The Customer acknowledges that:

12.1.1 all Intellectual Property Rights in the Data are the property of TalkLife or its licensors, as the case may be;

12.1.2 it shall have no rights in or to the Data other than the right to use them in accordance with the express terms of this Agreement; and

12.1.3 TalkLife or its licensors has or have made and will continue to make substantial investment in the obtaining, verification, selection, coordination, development, presentation and supply of the Data.

12.2 The Customer shall, and shall use all reasonable endeavours to procure that any necessary third party shall, at TalkLife's cost, promptly execute such documents and perform such acts as may reasonably be required for the purpose of giving full effect to this Agreement.

12.3 Any use of the Data by the Customer shall credit, wherever technically and commercially feasible, TalkLife, any licensor of TalkLife or any other source of the Data specified by TalkLife as the source of the Data.

12.4 Subject to clause 14.4, TalkLife undertakes to defend the Customer from and against any claim or action that the provision, receipt or use of the Data (wholly or in part) infringes any Intellectual Property Right of a third party (**IPR Claim**) and shall be responsible for any losses, damages, costs (including all legal fees) and expenses incurred by or awarded against the Customer as a result of, or in connection with, any such IPR Claim, provided that, if any third party makes an IPR Claim, or notifies an intention to make an IPR Claim against the Customer, the Customer shall:

12.4.1 give written notice of the IPR Claim to TalkLife as soon as reasonably practicable;

12.4.2 not make any admission of liability in relation to the IPR Claim without the prior written consent of TalkLife;

12.4.3 at TalkLife's request and expense, allow TalkLife to conduct the defence of the IPR Claim including settlement; and

12.4.4 at TalkLife's expense, co-operate and assist to a reasonable extent with TalkLife's defence of the IPR Claim.

12.5 If any IPR Claim is made, or in TalkLife's reasonable opinion is likely to be made, against the Customer, TalkLife may at its sole option and expense:

12.5.1 procure for the Customer the right to continue using, developing, modifying or retaining the Data (wholly or in part) in accordance with this Agreement;

12.5.2 modify the Data (wholly or in part) so that they cease to be infringing;

12.5.3 replace the Data (wholly or in part) with non-infringing items; or

12.5.4 terminate this Agreement immediately by notice in writing to the Customer and refund any Licence Fee paid by the Customer as at the date of termination (less a reasonable sum in respect of the Customer's use of the Data to the date of termination) on return of the Data and all copies of each of them.

12.6 Clause 12.4 constitutes the Customer's sole and exclusive remedy and TalkLife's only liability in respect of IPR Claims.

13 **WARRANTIES**

13.1 TalkLife warrants that it has the right to license the Data as specified in this Agreement.

13.2 Except as expressly stated in this Agreement, all warranties, conditions and terms, whether express or implied by statute, common law or otherwise are hereby excluded to the extent permitted by law.

13.3 Without limiting the effect of clause 13.2, TalkLife does not warrant that:

13.3.1 the Data will run on the Customer System;

13.3.2 the Data is accurate, complete, reliable, secure, useful, fit for purpose or timely; or

13.3.3 the Data has been tested for use by the Customer or any third party or that the Data will be suitable for or be capable of being used by the Customer or any third party.

14 **LIMITATION OF LIABILITY**

14.1 Neither party excludes or limits liability to the other party for:

14.1.1 fraud or fraudulent misrepresentation;

14.1.2 death or personal injury caused by negligence;

14.1.3 a breach of any obligations implied by section 12 of the Sale of Goods Act 1979 or section 2 of the Supply of Goods and Data Act 1982; or

14.1.4 any matter in respect of which it would be unlawful for the parties to exclude liability.

14.2 Subject to clause 14.1, no party shall in any circumstances be liable whether in contract, tort (including for negligence and breach of statutory duty howsoever arising), misrepresentation (whether innocent or negligent), restitution or otherwise, for:

14.2.1 any loss (whether direct or indirect) of profits, business, business opportunities, revenue, turnover, reputation or goodwill;

14.2.2 any loss or corruption (whether direct or indirect) of data or information;

14.2.3 loss (whether direct or indirect) of anticipated savings or wasted expenditure (including management time); or

14.2.4 any loss or liability (whether direct or indirect) under or in relation to any other contract.

14.3 Clause 14.2 shall not prevent claims, which fall within the scope of clause 14.4, for:

14.3.1 direct financial loss that are not excluded under any of the categories set out in clause 14.2.1 to clause 14.2.4; or

14.3.2 tangible property or physical damage.

14.4 Subject to clauses 14.1 and 14.2, each party’s total aggregate liability in contract, tort (including negligence and breach of statutory duty howsoever arising), misrepresentation (whether innocent or negligent), restitution or otherwise, arising in connection with the performance or contemplated performance of this Agreement shall in all circumstances be limited to a sum equal to the Licence Fee to be paid by the Customer to TalkLife.

## 15 TERM AND TERMINATION

15.1 This Agreement shall commence on the Effective Date. Unless terminated earlier in accordance with clauses 7, 15.2, or 17, this Agreement shall continue until completion of the Project.

15.2 Without prejudice to any rights that have accrued under this Agreement or any of its rights or remedies, either party may terminate this Agreement with immediate effect by giving written notice to the other party if:

15.2.1 the other party fails to pay any amount due under this Agreement on the due date for payment and remains in default not less than 14 days after being notified in writing to make that payment;

15.2.2 the other party commits a material breach of any material term of this Agreement (other than failure to pay any amounts due under this Agreement) and (if that breach is remediable) fails to remedy that breach within a period of 30 days after being notified in writing (including email) to do so;

15.2.3 the other party:

15.2.3.1 suspends, or threatens to suspend, payment of its debts;

15.2.3.2 is unable to pay its debts as they fall due or admits inability to pay its debts; or

15.2.3.3 (being a company) is deemed unable to pay its debts within the meaning of section 123 of the Insolvency Act 1986;

15.2.4 the other party commences negotiations with all or any class of its creditors with a view to rescheduling any of its debts, or makes a proposal for or enters into any compromise or arrangement with its creditors other than for the sole purpose of a scheme for a solvent amalgamation of that other party with one or more other companies or the solvent reconstruction of that other party;

15.2.5 a petition is filed, a notice is given, a resolution is passed, or an order is made, for or in connection with the winding up of that other party other than for the sole purpose of a scheme for a solvent amalgamation of that other party with one or more other companies or the solvent reconstruction of that other party;

15.2.6 an application is made to court, or an order is made, for the appointment of an administrator, or if a notice of intention to appoint an administrator is given or if an administrator is appointed, over the other party;

15.2.7 the holder of a qualifying floating charge over the assets of that other party has become entitled to appoint or has appointed an administrative receiver;

15.2.8 a person becomes entitled to appoint a receiver over the assets of the other party or a receiver is appointed over the assets of the other party;

15.2.9 a creditor or encumbrancer of the other party attaches or takes possession of, or a distress, execution, sequestration or other similar process is levied or enforced on or sued against, the whole or any part of the other party's assets and that attachment or process is not discharged within 14 days;

15.2.10 any event occurs or proceeding is taken with respect to the other party in any jurisdiction to which it is subject that has an effect equivalent or similar to any of the events mentioned in clause 15.2.3 to clause 15.2.9 (inclusive); or

15.2.11 the other party suspends or ceases, or threatens to suspend or cease, carrying on all or a substantial part of its business.

## 16 CONSEQUENCES OF TERMINATION

16.1 Any provision of this Agreement that expressly or by implication is intended to come into or continue in force on or after termination of this Agreement shall remain in full force and effect.

16.2 Termination or expiry of this Agreement shall not affect any rights, remedies, obligations or

liabilities of the parties that have accrued up to the date of termination or expiry, including the right to claim damages in respect of any breach of the Agreement which existed at or before the date of termination or expiry.

16.3 On expiry or termination of this Agreement for any reason, the Customer shall immediately pay any outstanding amounts owed to TalkLife under this Agreement.

16.4 On termination of this Agreement in accordance with clause 7, the licence granted to the Customer at clause 2 shall immediately terminate and the Customer must return or destroy (at TalkLife's option) all Data, information, software, and other materials provided to it by the other party in connection with this Agreement including Confidential Information.

16.5 On completion of the Project, the Customer party shall as soon as reasonably practicable return or destroy (as directed in writing by TalkLife) all Data, information, software, and other materials provided to it by TalkLife in connection with this Agreement including TalkLife's Confidential Information and the Customer shall ensure that all Data and Confidential Information is deleted (as far as reasonably technically possible) from the Customer System. For the avoidance of doubt, TalkLife acknowledges that such return or destruction shall not include the Customer's findings or research results which has been derived from the Data and that this shall not apply to any information contained, reflected or referred to in (i) any board minutes or other documents which either party, its consultants or financiers or professional advisers are required to retain under any applicable law or regulation or to comply with the rules of any regulatory body or authority, or (ii) any electronic back-up copies made automatically in the ordinary course of safe-guarding electronic records).

16.6 The Customer shall, on TalkLife's request, provide written confirmation (in the form of a letter signed by an authorised signatory) of compliance with clause 16 no later than 14 days after TalkLife's request.

17 **FORCE MAJEURE**

Neither party shall be in breach of this Agreement nor liable for delay in performing, or failure to perform, any of its obligations under this Agreement if such delay or failure result from events, circumstances or causes beyond its reasonable control. In such circumstances the affected party shall be entitled to a reasonable extension of the time for performing such obligations. If the period of delay or non-performance continues for [4] [weeks], the party not affected may terminate this Agreement by giving [30] [days'] written notice to the affected party.

18 **ASSIGNMENT**

18.1 This Agreement is personal to the Customer and it shall not assign, transfer, mortgage, charge, sub-contract, declare a trust of or deal in any other manner with any of its rights and obligations under this Agreement without the prior written consent of TalkLife (which is not to be unreasonably withheld or delayed).

18.2 The Customer confirms it is acting on its own behalf and not for the benefit of any other person.

18.3 TalkLife may at any time assign, transfer, mortgage, charge, sub-contract, declare a trust of or deal in any other manner with any of its rights and obligations under this Agreement without the consent of the Customer.

19 **WAIVER**

No failure or delay by a party to exercise any right or remedy provided under this Agreement or by law shall constitute a waiver of that or any other right or remedy, nor shall it preclude or restrict the further exercise of that or any other right or remedy. No single or partial exercise of any right or remedy shall preclude or restrict the further exercise of that or any other right or remedy.

20 **REMEDIES**

Except as expressly provided in this Agreement, the rights and remedies provided under this Agreement are in addition to, and not exclusive of, any rights or remedies provided by law.

21 **NOTICE**

21.1 Any notice given to a party under or in connection with this contract shall be in writing and shall be delivered by hand or by pre-paid first-class post or other next working day delivery service at its registered office (if a company) or its principal place of business (in any other case).

21.2 Any notice shall be deemed to have been received:

21.2.1 if delivered by hand, on signature of a delivery receipt or at the time the notice is left at the proper address; or

21.2.2 if sent by pre-paid first-class post or other next working day delivery service, at 9.00 am on the second day after posting [or at the time recorded by the delivery service.

21.3 This clause does not apply to the service of any proceedings or other documents in any legal action or, where applicable, any arbitration or other method of dispute resolution. For the purposes of this clause, "writing" shall include email.

22 **ENTIRE AGREEMENT**

22.1 This Agreement constitutes the entire agreement between the parties and supersedes all previous discussions, correspondence, negotiations, arrangements, understandings and agreements between them relating to its subject matter.

22.2 Each party acknowledges that in entering into this Agreement it does not rely on, and shall have no remedies in respect of, any representation or warranty (whether made innocently or negligently) that is not set out in this Agreement.

22.3 Each party agrees that it shall have no claim for innocent or negligent misrepresentation or negligent misstatement based on any statement in this Agreement.

23 **VARIATION**

Except as expressly provided in this Agreement, no variation of this Agreement shall be effective unless it is in writing and signed by the parties (or their authorised representatives).

24 **SEVERANCE**

24.1 If any provision or part-provision of this Agreement is or becomes invalid, illegal or unenforceable, it shall be deemed modified to the minimum extent necessary to make it valid, legal and enforceable. If such modification is not possible, the relevant provision or part-provision shall be deemed deleted. Any modification to or deletion of a provision or part-provision under this clause shall not affect the validity and enforceability of the rest of this Agreement.

24.2 If one party gives notice to the other of the possibility that any provision or part-provision of this Agreement is invalid, illegal or unenforceable, the parties shall negotiate in good faith to amend such provision so that, as amended, it is legal, valid and enforceable, and, to the greatest extent possible, achieves the intended commercial result of the original provision.

25 **NO PARTNERSHIP OR AGENCY**

25.1 Nothing in this Agreement is intended to, or shall be deemed to, establish any partnership or joint venture between any of the parties, constitute any party the agent of another party, or authorise any party to make or enter into any commitments for or on behalf of any other party.

25.2 Each party confirms it is acting on its own behalf and not for the benefit of any other person.

26 **THIRD-PARTY RIGHTS**

A person who is not a party to this Agreement shall not have any rights under the Contracts (Rights of Third Parties) Act 1999 to enforce any term of this Agreement.

27 **GOVERNING LAW AND JURISDICTION**

27.1 This Agreement and any dispute or claim (including non-contractual disputes or claims) arising out of or in connection with it or its subject matter or formation shall be governed by and construed in accordance with the law of England.

27.2 Each party irrevocably agrees that the courts of England and Wales shall have exclusive jurisdiction to settle any dispute or claim (including non-contractual disputes or claims) arising out of or in connection with this Agreement or its subject matter or formation.
